# Tectorial Membrane Injury, Frequently Identified in Adult Trauma Patients Who Undergo Occipital-Cervical Fusion for Craniocervical Instability

**DOI:** 10.7759/cureus.14254

**Published:** 2021-04-02

**Authors:** Peter Fiester, Dinesh Rao, Erik Soule, Matthew Jenson, Jeet Patel

**Affiliations:** 1 Neuroradiology, University of Florida College of Medicine, Jacksonville, USA; 2 Interventional Radiology, University of Florida College of Medicine, Jacksonville, USA

**Keywords:** trauma, craniocervical junction, craniocervical disassociation, atlantooccipital dislocation, magnetic resonance imaging, ligamentous injury

## Abstract

Background

In the absence of frank craniocervical dissociation, there is a lack of consensus regarding what patterns of craniocervical junction ligamentous injuries require occipital-cervical fusion. This study was undertaken to examine the integrity of the craniocervical junction ligaments and analyze clinical outcomes in patients who underwent occipital-cervical fusion for craniocervical junction injury.

Methods

Adult patients requiring occipital-cervical fusion were identified retrospectively utilizing keyword searches in cervical computed tomography and magnetic resonance imaging reports between 2012 and 2020 using Nuance mPower software (Nuance, Burlington, MA). The cervical magnetic resonance imaging examinations for these patients were reviewed for craniocervical ligamentous injury by two neuroradiologists. Descriptions of craniocervical junction injuries, demographic information, clinical history, surgical management, and global outcomes were recorded.

Results

Nine adult patients were identified with an acute, post-traumatic craniocervical junction injury requiring occipital-cervical fusion. All nine patients demonstrated a ligamentous tear in at least one of the four major craniocervical junction ligaments - the occipital condylar-C1 capsular ligaments, alar ligaments, tectorial membrane, and posterior atlantooccipital membrane. The tectorial membrane was the most commonly torn ligament followed by the alar ligament(s), posterior atlantooccipital membrane, and capsular ligament(s). There was wide variability in the number of major craniocervical junction ligaments torn, ranging from one ligament to all four ligaments. Four patients suffered persistent neurologic deficits following surgery.

Conclusion

Craniocervical injury is best evaluated by cervical magnetic resonance imaging. In the absence of overt craniocervical dissociation, we propose that an injury of the tectorial membrane in the adult population may indicate patients with significant craniocervical instability, possibly necessitating occipital-cervical fusion.

## Introduction

The craniocervical junction (CCJ) is a unique osteoligamentous structure that comprises the occipital condyles, skull base, first and second cervical vertebrae. The atlantooccipital joint, atlantoaxial joint, and several surrounding stabilizing ligaments between the skull base and C1-C2 are also incorporated within the CCJ. The CCJ must balance the need for maximum stability and protection of the brainstem, cervical cord, and surrounding vital neurovascular structures with enough mobility to allow ample neck flexion, extension, rotation, and lateral bending. The trauma of the CCJ is a rare and potentially life-threatening injury and ranges from isolated ligamentous and bony injuries to frank craniocervical dissociation (CCD) and closed-capitation injury [1,2]. CT is the modality of choice to evaluate for CCJ trauma since it readily depicts bony fractures and condylar-C1 joint subluxation and dislocation [3]. However, CT poorly depicts soft tissue injuries and does not directly evaluate the major stabilizing ligaments of the CCJ. Thus, in the absence of bony trauma or abnormal joint space widening at the CCJ, CT may underreport significant craniocervical trauma. This situation may have devastating clinical consequences since the early diagnosis of unstable craniocervical junction injury and treatment with spinal stabilization protect against worsening spinal cord injury and reduce the likelihood of neurologic deterioration [4].

MRI of the cervical spine is an increasingly utilized modality to evaluate for cervical cord and ligamentous injury since it allows direct inspection of the cervical cord and stabilizing ligaments of the CCJ. Increased use of cervical MRI is allowing a deeper understanding of craniocervical trauma as a spectrum of injury, ranging in severity from overt CCD with atlantooccipital dislocation to more subtle, isolated injuries to one or more of the craniocervical ligaments which may nevertheless predispose to the instability of the CCJ.

Previous cadaveric studies have suggested that four major ligaments - the atlantooccipital joint and enveloping capsular ligaments, alar ligaments, tectorial membrane (TM), and posterior atlantooccipital membrane complex (PAOMc) - provide the primary stabilization of the CCJ [5-8]. Multiple additional CCJ ligaments, including the anterior atlantooccipital membrane (AAOM), apical ligament, and the superior band of the cruciform ligament, play a nominal role in maintaining CCJ stability. Even more obscure CCJ ligaments, such as the superficial anterior atlantooccipital ligament, atlantodental ligament, and transverse occipital ligament, are not routinely visualized on cervical MRI [9-11]. Of note, the transverse band of the cruciform ligament, which tightly maintains the atlantoaxial joint, is not considered a craniocervical ligament since it does not maintain structural integrity between the cervical spine and skull base. Given the unique anatomic complexity of CCJ ligaments, paucity of literature regarding CCJ ligamentous injury patterns, and uncertainty about which ligaments provide the major stability for the CCJ, there remains little consensus in the radiologic and neurosurgical literature on the appropriateness of craniocervical fusion for CCJ osteoligamentous injuries especially in the absence of CCD with atlantooccipital dislocation.

Thus, a retrospective imaging analysis of the cervical MRI findings in patients requiring occipital-cervical fusion for acute craniocervical trauma with instability was performed in order to examine which major craniocervical ligaments were injured and evaluate for any patterns of ligamentous injury that may result in surgical fusion. The clinical presentation, management, and global outcome of our patient population were also analyzed.

## Materials and methods

A waiver of informed consent was granted to retrospectively examine the cervical MRI findings and electronic medical records for patients who suffered an acute, post-traumatic injury of the craniocervical junction requiring surgical fusion for craniocervical instability. Instability of the craniocervical junction was determined based on the neurosurgical review of patient clinical and radiologic findings in consensus with a certificate of additional qualification certified neuroradiologist. If present, subsequent occipital-cervical fusion was performed to restore craniocervical stability. The major craniocervical ligaments were defined as the atlantooccipital capsular joint and ligament, alar ligaments, tectorial membrane, and PAOMc. The minor craniocervical junction ligaments were defined as the AAOM, apical ligament, and superior band of the cruciform ligament. A keyword search of radiology reports using mPower software (Nuance, Burlington, MA) between March 2012 and June 2020 using the keywords "occipital-cervical fusion," "alar ligament," "tectorial membrane," "posterior atlantooccipital membrane," "craniocervical ligament tear/injury," and "atlantooccipital dissociation" was utilized in order to identify patients who required post-surgical craniocervical fusion for acute CCJ injury. There were 71 patients identified with major ligamentous injury at the CCJ, nine of which underwent occipital-cervical fusion.

MRI examinations were performed using the standard departmental protocols. MRI studies were performed on a 1.5 Tesla magnet with a head and neck coil (Avanto, Siemens, Munich, Germany). Slice thickness was 3 mm and sagittal T1, T2, and short tau inversion recovery (STIR) as well as axial T2, and T2 Multi-Echo Data Image Combination (MEDIC) sequences were obtained. All cervical MRI examinations were graded for their diagnostic quality and were reviewed by two experienced certificates of additional qualification certified neuroradiologists in consensus.

A thorough analysis of the major CCJ ligaments (capsular ligaments, alar ligaments, tectorial membrane, and PAOMc) was performed. Additionally, the integrity of the minor CCJ ligaments (AAOM, apical ligament, superior band of the cruciform ligament) was evaluated. A craniocervical junction ligament was considered injured if the normal thin T2 hypointense band was disrupted and demonstrated an increased T2 signal. The atlantooccipital capsular ligament was considered torn if there was an abnormal widening of the atlantooccipital joint space greater than 2.5 mm. In addition, the location of any TM injury was divided into "subclival" if the injury occurred inferior to the clivus, or "retroclival" if the tear occurred posterior to the clivus. The tectorial membrane was also considered injured if it demonstrated a noticeably decreased thickness (defined as less than half the thickness of an intact TM) on sagittal T2 weighted sequence and was classified as a "stretch" injury, or partial tear. TM tears were also graded based on the classification system developed by Fiester et al. [12]. Finally, the anterior longitudinal ligament (ALL), posterior longitudinal ligament (PLL), ligamentum flavum, and interspinous ligaments from C2-C6 were evaluated for injury. If present, fractures of the clivus, occipital condyles, and cervical spine were documented.

The presence of intracranial trauma, including diffuse axonal injury, hemorrhagic or non-hemorrhagic contusion, and extra-axial hemorrhage (including epidural, subdural, and subarachnoid hemorrhage) was documented along with the presence of cervical cord injury. Finally, patient electronic patient records were reviewed for the following: (1) age and sex of the patient, (2) mechanism of trauma, (3) type and length of cervical fusion, and (4) clinical outcome.

## Results

Nine adult patients (seven female and two male) ranging between the age of 21 years and 78 years (average 40 years old) were identified retrospectively with acute, post-traumatic CCJ injury requiring occipital cervical fusion for craniocervical instability (Table 1).

**Table 1:** Patients with clinical and radiologic findings of acute, post-traumatic craniocervical junction instability who underwent occipital-cervical fusion, the status of the major craniocervical junction ligaments on cervical MRI, the surgical management, and clinical outcome. TM = tectorial membrane, PAOMc = posterior atlantooccipital membrane complex, MVA = motor vehicle accident, F = female, M = male

No.	Age/sex	Mechanism of Injury	TM Injury Type	Alar ligament tear	Atlantooccipital joint (<2.5 mm)	PAOMc tear	Cervical fracture	Surgical fusion	Outcome
1	40/F	MVA	Type 3	R. alar	5 mm	Yes	C7 transverse process	Occiput – T2	Quadriplegic
2	44/F	MVA	Type 2a	L. alar	1 mm	No	C1	Occiput – C3	Parapalegic
3	30/M	Pedestrian vs motor vehicle	Type 2b	None	1 mm	No	None	Occiput – C2	Full recovery
4	21/F	Pedestrian vs motor vehicle	Type 2b	Bilateral	3 mm	No	C1	Occiput – C4	Parapalegic
5	35/F	MVA	Type 2b	L. alar	10 mm	No	C1	Occiput – C3	Right extremity spasticity
6	35/F	Pedestrian vs motor vehicle	Type 3	None	1 mm	Yes	C2	Occiput – C3	Full recovery
7	78/F	Fall	None	None	1 mm	Yes	C1 and C2	Occiput – C3	Full recovery
8	34/M	MVA	Type 2b	R. alar	1 mm	Yes	C2	Occiput – C2	Full recovery
9	33/F	MVA	Type 2b	None	1 mm	Yes	None	Occiput - C3	Full recovery

The tectorial membrane was injured in all but one patient (six patients demonstrated complete TM disruption - type 2 injury, and two patients demonstrated a partial tear - type 3 injury). Of the remaining major CCJ ligaments, six patients demonstrated a unilateral or bilateral alar ligament tear (five patients unilateral and one patient bilateral), five patients demonstrated a PAOMc tear, and three patients demonstrated an atlantooccipital capsular ligament tear. One patient demonstrated a tear in all four major CCJ ligaments; three patients demonstrated a tear in three major CCJ ligaments; three patients demonstrated a tear in two major CCJ ligaments; and, two patients demonstrated a tear in one major CCJ ligament (tectorial membrane and PAOMc, respectively). Of note, one patient who underwent occipital-cervical fusion after a fall injured only the PAOMc with associated fractures of C1 and C2.

Of the minor CCJ ligaments, all nine patients demonstrated a tear in the AAOM and eight patients demonstrated a tear in the apical ligament. The superior band of the cruciform ligament could not be directly identified separately from the tectorial membrane in our nine patients and therefore was not included in our investigation. Regarding the remaining cervical spine ligaments, four patients demonstrated a tear of the transverse band of the cruciform ligament, four patients demonstrated an ALL tear (all at C2-C3) and five patients demonstrated a ligamentum flavum tear (three at C1-C2, one at C6-C7, and one at C7-T1). No patients demonstrated a PLL tear.

Seven patients demonstrated a cervical spine fracture. One patient had a C1 arch fracture and type II dens fracture; three patients had isolated C1 arch fractures; two patients had isolated C2 lateral mass fractures; and, one patient had a left C7 transverse process fracture. Half the patients demonstrated intracranial hemorrhage, including either subarachnoid or subdural hemorrhage. No patients had a cervical cord contusion.

Eight of the injuries involved motor vehicle accidents, either primary motor vehicle accidents or pedestrians struck by motor vehicles. One patient suffered a fall. Two patients underwent occipital-C2 fusion, four patients underwent occipital-C3 fusion, one patient underwent occipital-C4 fusion, and one patient received occipital-T2 fusion. Length of hospital stay ranged between 10 days and 79 days (average 33 days). All patients survived, yet clinical outcomes were variable. Five patients demonstrated no persistent neurologic deficits, three patients suffered a chronic neurologic deficit (weakness and/or spasticity), and one patient was quadriplegic.

## Discussion

**Figure 1:**
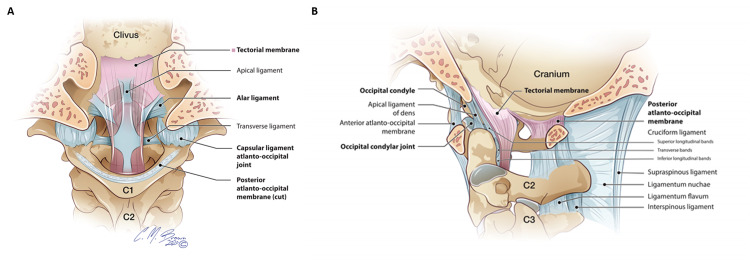
A and B: Coronal and sagittal illustrations of the major craniocervical junction ligaments. The tectorial membrane (pink) is the superiorly directed extension of the posterior longitudinal ligament located ventral to the thecal sac. The alar ligaments are paired ligaments that extend between the dens and medial occipital condyles. The atlantooccipital capsular ligaments envelop and reinforce the atlantooccipital joint. The posterior atlantooccipital membrane complex comprises the posterior atlantooccipital membrane (between the occiput and C1 posterior arch) and posterior atlantoaxial membrane (between posterior C1 arch and C2) which continues inferiorly as the ligamentum flavum. Original illustration used with permission from illustrator Chris Brown.

The tectorial membrane was the most common major CCJ ligament injury in our patient cohort. The TM was injured in all but one patient with six patients exhibiting a complete subclival disruption of the ligament (type 2 injury) and two patients exhibiting a TM “stretch” injury (type 3 injury) [12]. Anatomically, the TM is a strong ligament located posterior to the C2 dens that broaden out laterally at its cranial insertion along the posterior clivus before incorporating directly with the intracranial dura mater (Figure 1). 

The TM functions as a superiorly-directed extension of the PLL, which is located ventral to the spinal cord and inferior to the C2 level. Cadaveric descriptions of the TM describe it as firmly adherent to the skull base and C2 body, forming a ‘sling’ posterior to the dens, and attaching superiorly on the posterior clivus [13]. Whereas in pediatric patients the TM is typically stripped off the posterior clivus resulting in a venous retroclival epidural hematoma, the TM is most commonly fully disrupted along its subclival segment in adult patients [12,14]. 

**Figure 2:**
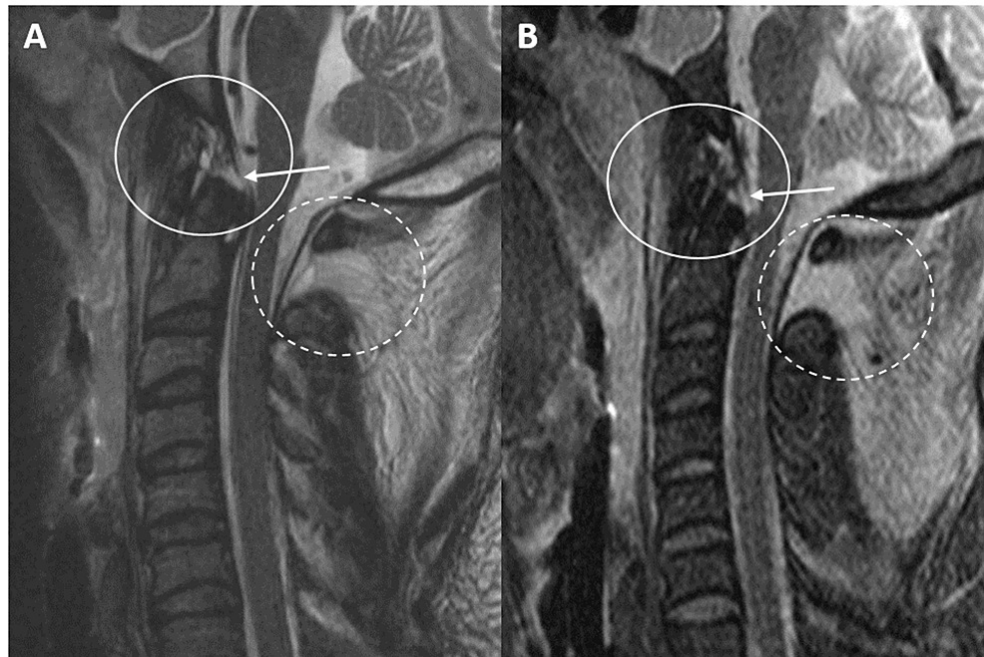
A and B: Sagittal T2 and STIR MRI sequences of the craniocervical junction and cervical spine demonstrate a subclival tectorial membrane tear (white arrows/circles) in a 34-year-old female status post motor vehicle collision. She subsequently underwent occipital-C4 posterior fusion for craniocervical instability. Additional tears of the anterior atlantooccipital membrane, apical ligament, posterior atlantooccipital membrane complex (dashed circle), and interspinous ligaments are also noted. STIR = short tau inversion recovery

Our findings suggest that the TM is vital in maintaining CCJ stability. With a significant enough hyperflexion-hyperextension force, the subclival TM may tear or become stretched thus disrupting the main stabilizing force between the central skull base and middle column of the cervical spine (Figure 2).

This results in abnormal mobility between the cranium and cervical spine and places the brain stem and upper cervical cord at risk for injury. Subsequently, a delay in recognition of TM injury may have devastating clinical consequences since several studies have demonstrated the importance of prompt recognition of CCJ instability and management with surgical fusion. Based on our study, tectorial membrane integrity is contributory in determining which adult patients with CCJ injury on cervical MRI are deemed, operative candidates. 

Two-thirds of our patients demonstrated an alar ligament tear (five patients unilateral and one patient bilateral). As a paired ligament extending superolateral from the dens to the medial surface of the occipital condyles, the alar ligaments hold the C2 dens upright and in close conjunction with the skull base and anterior C1 arch. Given the overall likelihood of alar ligament tear on MRI in patients who underwent surgical fusion, our findings support prior descriptions of the alar ligaments as a major stabilizing ligament of the CCJ [15,16]. This concept is important to recognize for two reasons. Whereas adult TM injuries are not directly visualized on cervical spine CT examinations, alar ligament tears may periodically present on CT as a small bony avulsion fracture from the occipital condyle or tip of the dens. Thus, recognition of an avulsion fracture in these locations on cervical spine CT should likely warrant a follow-up cervical MRI to evaluate the remaining major CCJ ligaments and exclude CCJ ligamentous injury patterns which may predispose to instability. Secondly, the importance of the alar ligaments in maintaining CCJ stability necessitates direct inspection of these ligaments on all cervical MRI examinations in the acute trauma setting. Given the relatively small size of the alar ligament, anatomic complexity of the CCJ, and potential for motion and artifact degraded imaging in the trauma setting, direct inspection of the alar ligaments may be challenging [17-19]. However, specifically-tailored MRI protocols in the setting of clinical or radiologic suspicion for CCJ injury, including both coronal T2-weighted imaging and multiplanar imaging, may help mitigate these challenges [20,21]. 

**Figure 3:**
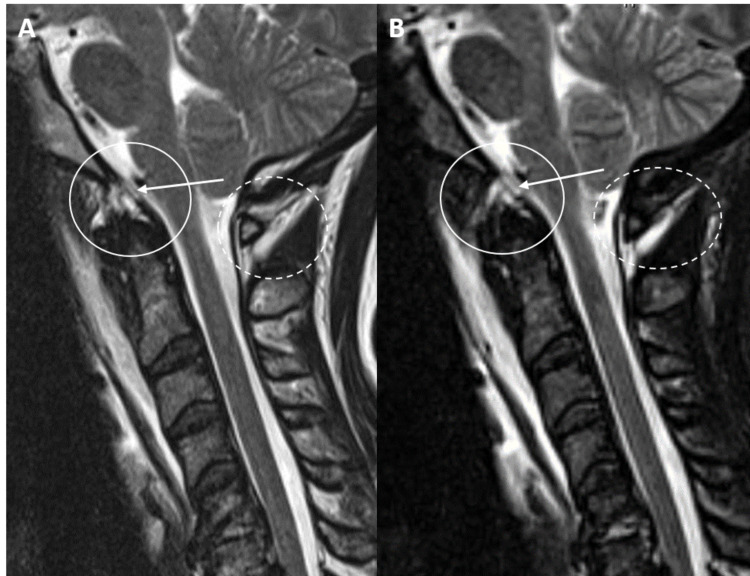
A and B: Sagittal T2 and STIR MRI sequences of the craniocervical junction and cervical spine demonstrate a subclival tectorial membrane tear (white arrows/circles) adjacent to the basion of the clivus in a 30-year-old male status post motor vehicle collision. Additional tear of the posterior atlantooccipital membrane complex is demonstrated (dashed circle). STIR = short tau inversion recovery

Five patients requiring surgical fusion demonstrated a PAOMc tear (Figure 3).

The PAOMc consists of the posterior atlantooccipital membrane (PAOM) and posterior atlantoaxial membrane (PAAM). The PAOM is a broad, fan-shaped midline ligament that runs between the posterior C1 arch and the posterior margin of the foramen magnum where it fuses directly with the occipital dura similar to the TM’s insertion with the retroclival dura. It serves as the superior extension of the PAAM, which extends between the C1 and C2 level, and subsequently continues as the ligamentum flavum inferior to the C2 level. The PAOMc helps prevent hyperflexion and impingement of the atlas on the cervicomedullary junction and is thought to function as a primary stabilizing structure of the posterior craniocervical junction.

Of note, only one-third of our patient cohort who underwent occipital cervical fusion for acute, post-traumatic CCJ instability demonstrated an atlantooccipital capsular ligament disruption with subsequent widening of the atlantooccipital joint greater than 2.5 mm. The atlantooccipital capsular ligament is a tough ligament that envelops and reinforces the integrity of the atlantooccipital joint - a mildly mobile, synovial joint that allows approximately 25 degrees of flexion/extension and five degrees of rotation [22-25]. It is a primary CCJ stabilizing structure. According to the neurosurgical literature, abnormal widening of the atlantooccipital joint space greater than 2.5 mm in adults on cervical spine CT examinations is the most sensitive imaging findings to predict craniocervical dissociation [26-28]. 

The fact that two-thirds of our patients demonstrated no widening of the atlantooccipital joint is interesting for several reasons. Recent neurosurgical literature has suggested that widening of the atlantooccipital joint space carries the highest sensitivity for significant CCJ injury - often referred to as craniocervical dissociation or atlantooccipital dislocation - and the subsequent surgical management of occipital-cervical fusion for stabilization. However, only one-third of our patients demonstrated overt capsular ligamentous disruption and joint widening at the atlantooccipital joint [29]. Thus, relying solely on atlantooccipital subluxation/dislocation on cervical spine CT to screen trauma patients for unstable CCJ injury may overlook significant CCJ trauma and instability. A high index of clinical and radiologic suspicion is warranted as to not overlook these serious injuries on clinical evaluation and while interpreting cervical CT examinations. This point reinforces that cervical MRI remains vital to exclude CCJ injury by allowing direct inspection of the major CCJ ligaments.

Our results demonstrated wide variability in the number of major CCJ ligaments torn on cervical MRI. Only one patient demonstrated a tear in all four major CCJ ligaments, three patients demonstrated three ligament tears, three patients demonstrated two tears, and two patients demonstrated only one major CCJ ligament tear (tectorial membrane and PAOMc, respectively). This variability underscores the lack of consensus on which ligaments are the major stabilizing structures of the CCJ and which injury phenotypes constitute CCJ instability requiring surgical fusion. It is important to note that there may be confounding clinical reasons to perform occipital-cervical fusion in some patients, such as clinical examination findings suggestive of CCJ instability if the patient cannot tolerate prolonged external bracing or has risk factors that may predispose to future cord injury (increased fall risk or underlying canal stenosis) [30]. Nevertheless, given the significant reduction in neck mobility related to occipital-cervical fusion surgery, there may be select patients with only one or two CCJ ligament tears that could benefit from more conservative treatment with external and temporary stabilization. Future research studies with a large sample size would be of benefit to further investigate this possibility.

The minor ligaments of the CCJ - namely the AAOM and apical ligaments - were also evaluated in our study. While these ligaments likely play a nominal role in maintaining CCJ stability, the majority of patients who underwent occipital cervical fusion did demonstrate tears in one or both of these ligaments. Thus, in patients who underwent fusion, there may be a higher prevalence of minor ligament tears. 

**Figure 4:**
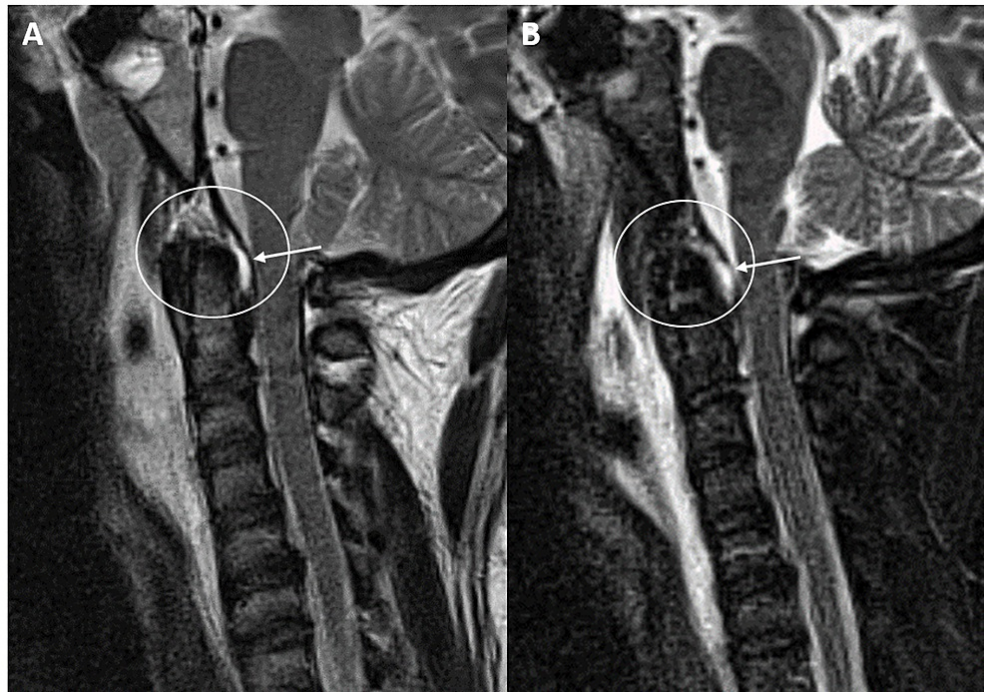
A and B: Sagittal T2 and STIR MRI sequences of the craniocervical junction and cervical spine demonstrate a subclival tectorial membrane tear posterior to the dens (white arrows/circles) near the ligamentous insertion in a 71-year-old female status post motor vehicle collision. Additional tears of the anterior longitudinal ligament at C1 and posterior atlantooccipital membrane complex are demonstrated. STIR = short tau inversion recovery

Finally, evaluation of the major stabilizing ligaments of the cervical spine proper was performed in our patient population. Four patients demonstrated an ALL tear and five patients demonstrated a ligamentum flavum tear (Figure 4). 

**Figure 5:**
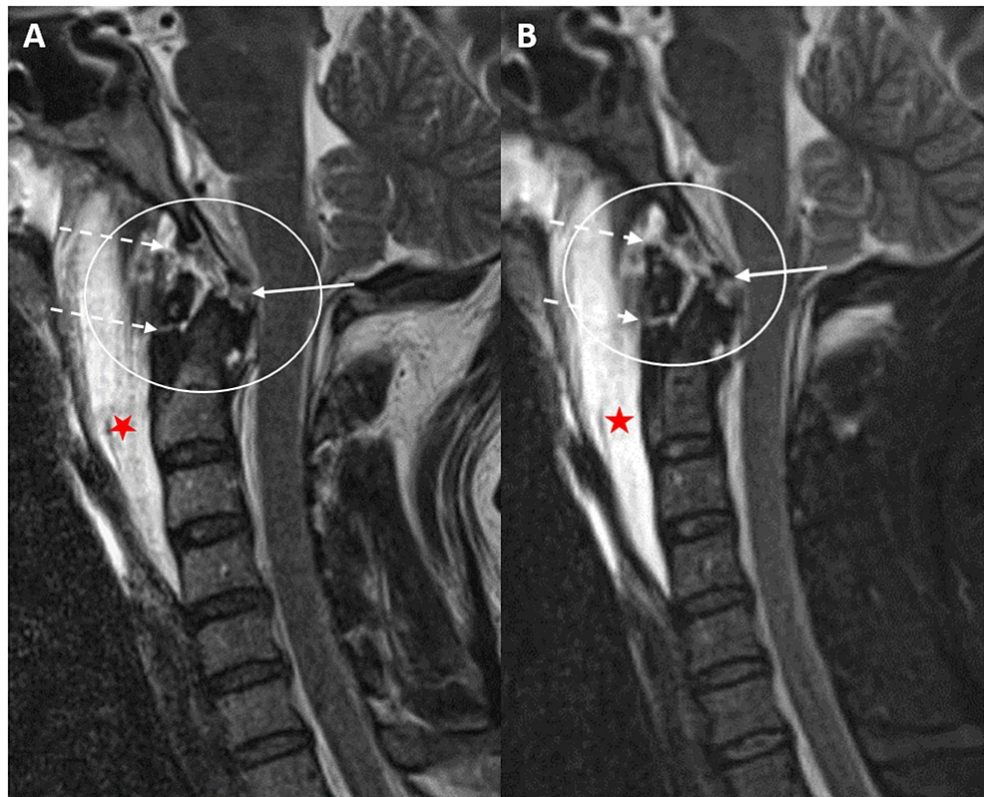
A and B: Sagittal T2 and STIR magnetic resonance imaging sequences of the craniocervical junction and cervical spine to demonstrate a subclival tectorial membrane tear (white arrow) in a 49-year-old male status post automobile vs. pedestrian incident. Additional tears of the anterior atlantooccipital membrane and anterior longitudinal ligament at C2 (dashed arrows) with a large prevertebral effusion (red star). STIR = short tau inversion recovery

No PLL injuries were present. Interestingly, all of the ALL tears occurred at the C2-C3 level and 60% of the ligamentum flavum tears occurred at the C2 level at the junction between the posterior atlantoaxial membrane and ligamentum flavum (Figure 5). 

Nearly 80% of patients underwent occipital-cervical fusion to at least the C3 level. Defining the overall extent of cervical osteoligamentous injury beyond the post-traumatic findings of the CCJ is crucially important as this not only supports the decision to surgically fuse these patients but may affect the overall extent of the surgical fusion.

All but one patient demonstrated a high-velocity injury consistent with the extreme hyperflexion-hyperextension force necessary to cause an unstable CCJ injury. Although all patients survived, nearly half the patients demonstrated persistent neurologic deficits and one patient was quadriplegic. Concomitant intracranial trauma was common and contributed to the neurologic deficits in these patients. 

The study had multiple limitations, including the small sample size obtained - although to our knowledge this is the only collection of patients with acute CCJ injury requiring operative fusion in the literature. Another limitation is the retrospective nature of the study, as a prospective study is required to prove a correlation between two entities. Selection bias may be a factor given that a control group was not studied (trauma patients who injured a major ligament of the CCJ but did not undergo occipital-cervical fusion). The search criteria may not have captured all of these patients, limiting the power of the study. Three of the nine patients included in the study had frank evidence of AOD with widening at the atlantooccipital joint. Other limitations include the possibility of interpreter error and variation in the quality/type of examination performed. Finally, prior literature has suggested that the evaluation of the smaller ligaments of the CCJ, such as the apical ligament, may be difficult to consistently visualize. Future directions include a study with larger sample size, to prospectively include patients who suffer a ligamentous injury at the CCJ and may or may not undergo occipital-cervical fusion. Comparative analysis and correlation with dynamic imaging such as flexion/extension lateral radiographs to assess motion around the CCJ may allow a more definitive conclusion regarding which phenotypes of ligamentous injury may result in craniocervical instability, and ultimately, occipital-cervical fusion. A multidisciplinary approach to include increased length of follow-up with more detailed descriptions of long-term neurologic sequelae may provide additional insight.

## Conclusions

An unstable craniocervical junction injury is a rare and life-threatening condition that, if survivable, may be treated with occipital-cervical fusion to prevent further neurologic deterioration and death. In the absence of overt craniocervical dissociation and widening of the atlantooccipital joint space, there is a lack of consensus in which patterns of craniocervical ligamentous injuries on cervical MRI predispose to instability requiring occipital cervical fusion. Based on our research, injury to the tectorial membrane was prevalent in patients who underwent occipital-cervical fusion. A thorough understanding of the anatomy, imaging findings of CCJ ligamentous injury on MRI, and contribution of each ligament toward maintaining craniocervical integrity is critical to properly evaluate and manage patients with CCJ trauma.
